# A Poroelastic Approach for Modelling Myocardial Oedema in Acute Myocarditis

**DOI:** 10.3389/fphys.2022.888515

**Published:** 2022-07-04

**Authors:** Wesley de Jesus Lourenço, Ruy Freitas Reis, Ricardo Ruiz-Baier, Bernardo Martins Rocha, Rodrigo Weber dos Santos, Marcelo Lobosco

**Affiliations:** ^1^ Graduate Program on Computational Modelling, Federal University of Juiz de Fora, Juiz de Fora, Brazil; ^2^ Department of Computer Science, Institute of Exact Sciences, Federal University of Juiz de Fora, Juiz de Fora, Brazil; ^3^ School of Mathematics and Victorian Heart Institute, Monash University, Melbourne, VIC, Australia; ^4^ Research Core on Natural and Exact Sciences, Universidad Adventista de Chile, Chillán, Chile; ^5^ World-Class Research Center “Digital Biodesign and Personalized Healthcare”, Sechenov First Moscow State Medical University, Moscow, Russia

**Keywords:** myocarditis, oedema formation, computational modelling, computational immunology, large-strain poroelasticity

## Abstract

Myocarditis is a general set of mechanisms that manifest themselves into the inflammation of the heart muscle. In 2017, more than 3 million people were affected by this disease worldwide, causing about 47,000 deaths. Many aspects of the origin of this disease are well known, but several important questions regarding the disease remain open. One of them is why some patients develop a significantly localised inflammation while others develop a much more diffuse inflammation, reaching across large portions of the heart. Furthermore, the specific role of the pathogenic agent that causes inflammation as well as the interaction with the immune system in the progression of the disease are still under discussion. Providing answers to these crucial questions can have an important impact on patient treatment. In this scenario, computational methods can aid specialists to understand better the relationships between pathogens and the immune system and elucidate why some patients develop diffuse myocarditis. This paper alters a recently developed model to study the myocardial oedema formation in acute infectious myocarditis. The model describes the finite deformation regime using partial differential equations to represent tissue displacement, fluid pressure, fluid phase, and the concentrations of pathogens and leukocytes. A sensitivity analysis was performed to understand better the influence of the most relevant model parameters on the disease dynamics. The results showed that the poroelastic model could reproduce local and diffuse myocarditis dynamics in simplified and complex geometrical domains.

## 1 Introduction

Inflammation or inflammatory process is a direct response of the immune system to invaders, also called pathogens. When the pathogen breaks our first barrier of defence, formed by the tissue and mucous, and enters the tissue, it begins to propagate through a mechanism that favours its rapid replication (in the case of viruses) or its reproduction (in the case of bacteria). The innate immune system will be ready to refrain from such replication/reproduction. Some cells that compose them, such as leukocytes, can detect the presence of pathogens using receptors on their surface. These receptors can recognise the presence of substances that are not naturally present in the body. Once the invasion is detected, the leukocyte start the production of pro-inflammatory cytokines to recruit more cells to fight against the pathogen. The pro-inflammatory cytokines play distinct roles. For example, some of them act as a chemoattractant, recruiting more cells to the site where the pathogen was found. Other pro-inflammatory cytokines facilitate the migration of leukocyte from the bloodstream, where most of them are located, to the tissue by increasing the permeability of blood vessels. However, this increased permeability allows also plasma to leave the bloodstream. This process favours an accumulation of plasma and interstitial fluid in the region, characterising local oedema.

The formation of oedema on the tissue can impact the functionality of an organ. For example, myocarditis has been associated with SARS-CoV-2 respiratory infection (Luetkens et al., 2020; Sala et al., 2020). In this disease, the inflammation of the myocardium tissue can reduce the capacity of the heart to pump blood as well as can cause arrhythmia, *i.e.*, irregularities in the heartbeat. Myocarditis can be caused not only by SARS-CoV-2 but also by other pathogens (infectious myocarditis), allergens (immune-mediated myocarditis), and drugs (toxic myocarditis) (Caforio et al., 2013). Although multiple agents can cause myocarditis, viral infections are most common (Caforio et al., 2013). The literature reports that the number of myocarditis cases in 2017 was 3,071,000, which represents an increase of about 60% from 1990 (Wang et al., 2021). The disease caused about 47,000 deaths in 2017, against about 27,000 in 1990 (Wang et al., 2021). Myocarditis affects individuals of all ages (Wang et al., 2021): its prevalence is higher in the young (Caforio et al., 2013), but most of the deaths are observed in elderly individuals (Wang et al., 2021). Since these numbers were collected before the COVID-19 pandemic, they may increase even more.

Although the causes of myocarditis are clear, there are many open questions regarding the disease, primarily related to the reasons that some patients recover, some of them without any injury in the infected area, while others die (Tschöpe et al., 2020). Another question is why some patients develop a local inflammation while others develop a diffuse one. In other words, for some patients, the inflammation and the associated oedema are located in a single portion of the myocardium, while for other patients, they spread across the heart. The role of the pathogen and the immune system in the progression of the disease is still under discussion, which in turn impacts the use of best treatment strategies (Tschöpe et al., 2020).

This paper studies the oedema formation during acute myocarditis using a poroelastic myocardium model. The mathematical model is based on the one presented in a previous work (Barnafi et al., 2021), which developed a robust and efficient finite element formulation and preconditioner for its numerical solution. In the present work, we focus on a sensitivity analysis of the model parameters to identify those that can reproduce the formation of local or diffuse myocardial oedema. Numerical studies are presented for a one-dimensional case and a left ventricular geometry.

Mathematical models for describing myocarditis dynamics are rare in the literature. van der Vegt et al. (2022) proposed a mathematical model to represent autoimmune myocarditis and the effects of immune checkpoint inhibitors on the development of the disease. Their results show the importance of the presence of immune checkpoint inhibitors to the development of autoimmune myocarditis. This work differs from van der Vegt et al. (2022) in many aspects, starting with its focus on infectious myocarditis. Models for Interstitial fluid pressure (IFP) dynamics can be found in the literature (Phipps and Kohandel, 2011; Cattaneo and Zunino, 2014; Jain et al., 2014). The first mathematical model that combines IFP dynamics and immune response was first suggested in our previous studies (Reis et al., 2016a,b). The classical theory of poroelasticity mechanics of a fluid-saturated porous media that was first proposed in 1941 (Biot, 1941) has been widely used in several studies to include tissue deformation (Berger et al., 2016; Selvadurai and Suvorov, 2016; Suvorov and Selvadurai, 2016). Three other works proposed general models in one spatial dimension (Reis R. F. et al., 2019) and a two-dimensions (Reis et al., 2018; Reis et al., 2019 RF.) that couple the immune system response with poroelasticity theory. The models were used to describe inflammatory oedema formation by also including the modelling of fluid accumulation.

This work is organised as follows. Section 2 present the methods, specially the mathematical model used to represent the formation of oedema and its computational implementation. Next, Section 3 presents the simulations performed and their results. Section 4 presents the discussion about the results obtained. Finally, Section 5 draws our conclusions and plans for future works.

## 2 Methods

In this section we present the mathematical model and numerical methods used to describe acute myocarditis and the formation of oedema in cardiac tissue. First, we describe the model assumptions. Next, the poroelastic component of the model is presented. Then, pathogen-leukocyte dynamics of the immunologic system are introduced. Finally, the couplings between the components of the model are addressed.

### 2.1 Model Assumptions

This section presents the simplifying hypotheses that guided the development of the mathematical model. Such simplifications focus on the mechanisms of oedema formation, a multi-physics phenomenon involving the coupling of the poroelasticity of the myocardium and the immune-system dynamics, allowing us to concentrate on the aspects of either local or diffuse oedema formation processes.

Oedema occurs when an excessive fluid volume accumulates in the tissue, both inside and outside the cells. In this study, we consider when it occurs in the space between cells, *i.e.,* in the interstitium, and for this reason, it is called interstitial, or extracellular, oedema. Oedema can result from many distinct causes, from the use of medications, the final stages of pregnancy, or as a consequence of diseases. This paper models oedema caused by an inflammatory process. One characteristic of the inflammatory process is an increase in capillary permeability followed by an increase in capillary filtration. So, the model considers that corporal fluid balance is affected by an invading pathogen triggering the inflammatory process. Although the inflammatory process and its resolution involve many proteins and cells of the immune system, this paper concentrates on some innate immune system cells, the leukocytes, i.e., the white blood cells involved in the defence against pathogens. These types of cells include macrophages, dendritic cells, neutrophils, eosinophils, T cells, B cells, and natural killer cells, which can target many pathogens, such as fungi, viruses, bacteria and large parasites. In our simplification, we consider that pathogens and leukocytes are located in a poroelastic medium saturated with interstitial fluid, representing the living tissue. We also assume that leukocytes can remove pathogens from the interstitium using, for example, phagocytosis. We also consider that pathogens can reproduce/replicate in some way. After leukocytes remove pathogens from the tissue, they start the resolution of inflammation, removing all recruited cells from tissue, *i.e.*, returning tissue to its homeostasis. From the immune system perspective, the complete resolution of inflammation was not modelled: the model does not take into account the removal of leukocytes from tissue.

We also consider that the tissue represents the myocardium, although the model is general enough to represent other types of tissues. The active behaviour of the cardiac muscle was not considered in this work. Likewise, we do not include electrophysiology or other mechanisms, such as mechanical interactions between the heart and surrounding tissue/organs. The main difficulty in dealing with such phenomena in the present poroelastic model would be the differences in timescales (Chabiniok et al., 2016). Cardiac electrophysiology and heart contraction have fast dynamics compared to the slower timescales of oedema formation, which is the main focus of the present work.

We have previously considered tissue orthotropy in Barnafi et al. (2021). In the present study, left ventricular loadings are substantially simplified in our model due to the timescale of oedema formation. Here, we consider the myocardium tissue to be passive. In addition, while myocardial fibre architecture plays a significant role in other regimes, we decided to adopt a more straightforward isotropic approach for the present investigation of oedema formation.

Only part of the heart is modelled, the left ventricle (LV), and it is assumed that no displacement occurs in its base. Although not realistic (Pfaller et al., 2019), assuming zero displacements in the base has been used as typical boundary conditions for mechanical simulations of the LV (see, *e.g.*, Campos et al., 2019; Genet et al., 2014; Peirlinck et al., 2019 and the references therein).

### 2.2 Poroelastic Component of the Model

Followisng Barnafi et al. (2021), we assume the myocardium as a poroelastic medium saturated with interstitial fluid where two species, a non-specific pathogen (*c*_*p*_) and leukocytes (*c*_*l*_), interact and are transported within the interstitial fluid. Thus, we consider a domain $\Omega \subset R^{d}$ , with *d* = 1, 3 representing the volume occupied by a deformable porous structure in its reference configuration. We denote by **n** the normal vector on the boundaries *∂*Ω. A material particle is identified in the undeformed configuration *Ω* by its position **x**, whereas $u:\Omega \to R^{d}$ denotes the displacement field that defines the new position **x** + ***u*** in the deformed configuration. We denote by **F** the deformation gradient tensor given by **F** = **I** + Grad ***u***, where **I** is the identity tensor. We consider *J* = det**F**, and $C=F^{t}F$ , which is the right Cauchy-Green strain tensor. Here and in the sequel, the superscript ${\left(\cdot \right)}^{t}$ indicates the matrix transpose operator. The symbols grad and Grad will denote the gradient of scalar and vector quantities, taken with respect to the material coordinates. Similarly, Div and ***Div*** are the divergence operators for vector and tensor fields, respectively.

The flow through the porous tissue can be described by Darcy’s law. To describe the poroelastic behaviour of the tissue, the Biot formulation under the large strain regime is considered (see, e.g., MacMinn et al., 2016). Let the total stress be defined by the first Piola-Kirchhoff stress tensor **P**, which is given by

$$P=P_{\text{eff}}-\alpha pJF^{-t},$$ (2.1)

where **P**_eff_ is the effective stress tensor, *α* is the Biot-Willis modulus, and *p* is the pore fluid pressure. We consider the neo-Hookean model for the effective stress tensor, which results in

$$P_{\text{eff}}=\mu_{s}\left(F-F^{-t}\right)+\lambda_{s}\;\text{ln}\left(J\right)F^{-t},$$ (2.2)

where $\mu_{s}=\frac{E}{2\left(1+\nu \right)}$ and $\lambda_{s}=\frac{E\;\nu }{\left(1+\nu \right)\left(1-2\nu \right)}$ are the Lamé parameters defined in terms of Young’s modulus (*E*) and Poisson’s ratio (*ν*).

We consider the mixture to be saturated, that is, *ϕ*_*f*_ + *ϕ*_*s*_ = 1, where *ϕ*_*s*_ is the volume fraction of the solid phase. Further, we also assume that volume changes of the solid constituent are negligible, since volumetric stiffness of the solid is substantially smaller than the constituent [a reasonable assumption for soft living tissues (Zheng et al., 2020)]. Therefore, the following equation is considered

$$J=1+\phi_{f}-\phi_{0},$$

where *ϕ*_*f*_ − *ϕ*_0_ is the nominal porosity exchange in the reference configuration, and *ϕ*_0_ is the initial fluid phase. Note that this equation accounts for the material incompressibility of the constituents. It results from considering constant mass density for both phases (and nominal solid fraction being constant). However, as highlighted in MacMinn et al. (2016), this does not restrict the compression of the skeleton since the pore volume can change locally, implying modifications in the macroscopic mass density of the poroelastic material.

The proposed model is composed of five equations. Three of them define the poroelastic subsystem in terms of the displacement of the solid **u**, the fluid pressure *p* and the fraction of fluid phase *ϕ*_*f*_ as follows:

$$-\mathrm{Div}\left[P_{\text{eff}}-\alpha pJF^{-t}\right]=\rho_{s}bin\Omega \times (\left(0,t_{\text{final}}\right]\left.\right],$$ (2.3a)

$$\rho_{f}\frac{D}{Dt}\phi_{f}-\frac{1}{J}\mathrm{Div}\left(\phi_{f}\rho_{f}JF^{-1}\frac{\kappa }{\mu_{f}}F^{-t}gradp\right)=\rho_{f}\ell in\Omega \times (\left(0,t_{\text{final}}\right]\left.\right],$$ (2.3b)

$$J-\phi_{f}=1-\phi_{0}in\Omega \times (\left(0,t_{\text{final}}\right]\left.\right],$$ (2.3c)

where *α* is the Biot-Willis modulus, *ρ*_*s*_ is the solid phase density, ***b*** represents body forces (which are neglected in this work), *ρ*_*f*_ is the fluid phase density, $\frac{D}{Dt}$ represents the material derivative, *μ*_*f*_ is the dynamic viscosity, and ***κ*** is the hydraulic conductivity tensor described by ***κ*** = *κ*_0_ **I**, where *κ*_0_ is the absolute permeability. We remark that ***κ*** is defined in the spatial frame, where permeability measurements are taken (Wirth et al., 2014; Choo, 2018). Finally, *ℓ* is a distributed term that considers the exchange of fluid between the interstitial space, blood capillaries, and lymphatic capillaries, and is described next.

### 2.3 Immune System Component of the Model

To describe the immune system component of the model, we consider two species transported within the interstitial fluid in the poroelastic domain. We denote their concentrations by *c*_*p*_ and *c*_*l*_ representing pathogens and leukocytes, respectively.

**FIGURE 1:**
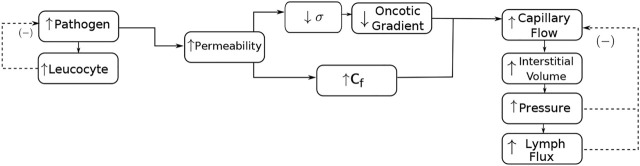
Interaction of the species and the poroelastic dynamics for modelling an inflammatory oedema formation. The increase in the pathogen concentration is responsible for an increase in leukocyte concentration. Leukocytes start the production of cytokines (not shown in the figure), which increases the permeability of the endothelium. The increase in permeability is manifested by the reduction in the oncotic reflection coefficient (*σ*) and the increase in hydraulic conductivity (*C*_*f*_). The reduction in the oncotic reflection coefficient also reduces the oncotic gradient. The increase in permeability also increases the resulting fluid interstitial exchange by blood capillaries (capillary flow), interstitial volume, pressure, and lymphatic flow. On the other hand, the increase in pressure and lymphatic flow results in negative feedback to the resulting capillary flow, tending to reduce it. The increase in leukocytes tends to reduce the presence of pathogens.

The inflammatory process is characterised by an increase in capillary permeability followed by an increase in capillary filtration. According to Scallan et al. (2010), these increases are manifested both by the reduction in the oncotic reflection coefficient (*σ*) and by the increase in the hydraulic conductivity (*C*_*f*_). This chain of events is illustrated in the flowchart in Figure 1. Therefore, the increase in permeability also leads to an increase in the resulting flow, interstitial volume, interstitial fluid pressure (*p*) and lymphatic flow. On the other hand, the increase of pressure and lymphatic flow results in negative feedback to the capillary reabsorption flow, tending to reduce it until the equilibrium is achieved.

An inflammatory reaction is triggered by the immune system when a pathogen enters the body. The objective is to protect the infection site against the antigen and control it. This process starts with the antigen presenting cells (APCs). These cells release pro-inflammatory cytokines, phagocyte the pathogen, and take it for presentation to the specialised adaptive cells. This cascade of events triggers the inflammation process and signals to the immune system the necessity for more defence cells (represented by leukocytes) in the infection site. This chain of events is simplified in our model as the reaction term for leukocytes *r*_*l*_, given by:

$$r_{l}=r_{l}\left(\phi_{f},c_{p},c_{l}\right)=\lambda_{pl}c_{p}c_{l},$$ (2.4)

where *λ*_*pl*_ denotes the leukocyte migration rate.

Pathogen reproduction/replication is assumed to be exponential with a rate of *r*_*p*_. Pathogen decay is due to a natural decay with rate *d*_*p*_ or due to its phagocytosis by a leukocyte (*λ*_*lp*_ *c*_*l*_ *c*_*p*_), resulting in the following reaction term:

$$r_{p}=r_{p}\left(\phi_{f},c_{p},c_{l}\right)=\phi_{f}\left(r_{p}-d_{p}-\lambda_{lp}c_{l}\right)c_{p},=\phi_{f}\left(\gamma_{p}-\lambda_{lp}c_{l}\right)c_{p},$$ (2.5)

where *γ*_*p*_ = *r*_*p*_ − *d*_*p*_ is the resulting pathogen reproduction rate and *λ*_*lp*_ is the leukocyte phagocytosis rate.

We consider the dynamics of pathogens (*c*_*p*_) and leukocytes (*c*_*l*_) written in the material configuration for the coupling with the poroelastic part and to take into account the mechanical feedback (Colli Franzone et al., 2016; Choo, 2018). The following equations are used to describe this:

$$\frac{D}{Dt}\left(\phi_{f}c_{p}\right)-\frac{1}{J}\mathrm{Div}\left(\phi_{f}JF^{-1}D_{p}F^{-t}Gradc_{p}\right)=r_{p}\;\;in\Omega \times \;(\left(0,t_{\text{final}}\right]\left.\right],$$ (2.6a)

$$\frac{D}{Dt}\left(\phi_{f}c_{l}\right)-\frac{1}{J}\;\mathrm{Div}\left(\phi_{f}JF^{-1}D_{l}F^{-t}Gradc_{l}-\chi \phi_{f}c_{l}JF^{-1}F^{-t}Gradc_{p}\right)=r_{l}\;\;in\Omega \times \;(\left(0,t_{\text{final}}\right]\left.\right],$$ (2.6b)

where **D**_*p*_ and **D**_*l*_ are diffusion tensors for pathogens and leukocytes, respectively; *r*_*p*_ and *r*_*l*_ are the reaction terms for pathogens and leukocytes, respectively; and *χ* is the chemotaxis coefficient. In particular, we assumed the pathogens and leukocytes diffusion tensors to be isotropic and represented by **D**_*p*_ = *d*_*p*_ **I** and **D**_*l*_ = *d*_*l*_ **I**, respectively, where *d*_*p*_ and *d*_*l*_ are the corresponding diffusion coefficients.

### 2.4 Coupling Term for Capillary Exchange

The first term of *ℓ* models the influence of blood capillaries [denoted by capillary flow in Figure 1) and is based on Starling equation (Starling, 1896)], whereas the second one represents the lymphatic flow, based on the Hill equation (Keener and Sneyd, 1998). Therefore, the interstitial fluid exchange term *ℓ* is given by:

$$\ell \left(p,c_{p}\right)=C_{f}\left(c_{p}\right)\left[p_{c}-p-\sigma \left(c_{p}\right)\left(\pi_{c}-\pi_{i}\right)\right]-q_{0}\left[1+\frac{v_{\max}{\left(p-p_{0}\right)}^{n}}{k_{m}^{n}+{\left(p-p_{0}\right)}^{n}}\right],$$ (2.7)

where

$$C_{f}\left(c_{p}\right)=\left(S/V\right)L_{p0}\left(1+c_{bp}c_{p}\right),\;and\;\sigma \left(c_{p}\right)=\sigma_{0}{\left(1+c_{bp}c_{p}\right)}^{-1}.$$

The remaining coefficients are the following: normal lymph flow (*q*_0_), capillary pressure (*p*_*c*_), oncotic pressure (*π*_*c*_), interstitial oncotic pressure due to plasma protein (*π*_*i*_), maximum lymph flow (*v*_max_), half-life pressure for lymphatic flow (*k*_*m*_), Hill coefficient (*n*), normal interstitial fluid pressure (*p*_0_), tissue wall capillary hydraulic permeability (*L*_*p*0_), pathogen influence on permeability (*c*_*bp*_) and area per unit of volume (*S*/*V*). It is worth noting that the oncotic gradient in Eq. 2.7 is represented by the term *σ*(*π*_*c*_ − *π*_*i*_).

### 2.5 Initial and Boundary Conditions

To close the system of Eqs 2.3a, 2.3b, 2.3c, 2.6a, and 2.6b, it is necessary to establish the initial and boundary conditions under which the problem is defined. Initially, the system will have the following configuration:

$$\begin{array}{c} \phi_{f}\left(x,0\right)=\phi_{0},\;\text{in}\;\Omega ,\;c_{l}\left(x,0\right)=0.003,\;\text{in}\;\Omega ,\\ c_{p}\left(x,0\right)=0.001,\;\text{in}\;\Omega_{c_{p}},\;c_{p}\left(x,0\right)=0.0,\;\text{in}\;\Omega \backslash \Omega_{c_{p}}, \end{array}$$

where $\Omega_{c_{p}}$ is the region where the infection begins. The following boundary conditions are considered:

$$u=0\;\text{on}\Gamma_{\text{base}}\times \left(0,t_{\text{final}}\right],$$ (2.8a)

$$\phi_{f}JF^{-1}\frac{\kappa \left(\phi_{f}\right)}{\mu_{f}}F^{-t}gradp\cdot n=0\;on\partial \Omega \times \left(0,t_{\text{final}}\right],$$ (2.8b)

$$Pn=0\;\text{on}\Sigma_{\text{endo}}\times \Sigma_{\text{epi}}\times \left(0,t_{\text{final}}\right],$$ (2.8c)

$$\phi_{f}JF^{-1}D_{p}F^{-t}gradc_{p}\cdot n=0\;\text{on}\partial \Omega \times \left(0,t_{\text{final}}\right],$$ (2.8d)

$$\left[\phi_{f}JF^{-1}D_{l}F^{-t}gradc_{l}-\chi \phi_{f}c_{l}JF^{-1}F^{-t}gradc_{p}\right]\cdot n=0\;\text{on}\partial \Omega \times \left(0,t_{\text{final}}\right],$$ (2.8e)

where *p*_0_ is a prescribed pressure on the epicardial surface Σ_epi_, Σ_endo_ denotes the endocardial surface, and Γ_base_ denotes the basal surface of the LV.

### 2.6 Numerical Implementation

To obtain a numerical approximation of the coupled model presented in the previous section we used the finite element method (FEM). The FEniCS software library (Langtangen and Logg, 2017) was used to obtain the numerical approximation of the variational formulation of the governing equations. Due to the nonlinear nature of the model, the Newton-Raphson method was used for the solution. In particular, the models were discretised using tetrahedral elements and time-derivatives approximated by backward Euler’s method using a monolithic approach. A mixed finite element formulation was used for the numerical approximation of the fields (***u***, *p*, *c*_*p*_, *c*_*l*_, *ϕ*_*f*_). In particular, the pair (***u***, *ϕ*_*f*_) was approximated using the MINI element (Arnold et al., 1984), quadratic Lagrangian elements were used for approximating *c*_*p*_ and *c*_*l*_, while *p* was approximated with linear Lagrangian elements. For the Newton-Raphson iterative algorithm, a tolerance of 10^–6^ was used. Computer simulations were carried out in a personal laptop equipped with an Intel(R) Core(TM) i7-9750H processor and 16 GB of RAM running on the Ubuntu Linux 20.04 LTS operating system. The entire code was written in *Python* and executed using the following versions in *Python* version 3.8.10 and FEniCS library version 2019.2. The results were post-processed with the Paraview software for visualisation.

## 3 Results

This section presents the results obtained by the numerical solution of the model, divided into dynamics over time, three-dimensional simulations of oedema formation, and a sensitivity analysis of the model. The first set of numerical experiments presents the dynamics of pathogens, leukocytes, pressure, phase and displacement over time in a one-dimensional domain. The idea is to understand their dynamics and relationships during the oedema formation. The second set of numerical experiments presents the results of the simulations in a three-dimensional representation of the LV using a ventricular geometry segmented from patient-specific images (Warriner et al., 2018). Finally, the last experiment presented in this section attempts to identify the impacts of the relevant model parameters in the output, *i.e.*, in the oedema formation. To simplify the approach, this analysis was carried out in a one-dimensional version of the model. For all the mentioned sets of numerical experiments we present separate results for local and diffuse myocarditis.

The parameter values used in all simulations are those presented in Tables 1, 2, which were used to simulate the local and diffuse myocarditis. The regions containing an initial concentration of pathogens, responsible for triggering the inflammatory response and dynamics of the poroelastic model, were defined as follows: for the one-dimensional simulations, $\Omega_{c_{p}}=3.8\leq x\leq 4.2$ , and for the LV simulations, $\Omega_{c_{p}}={\left(x-0.13047\right)}^{2}+{\left(y-3.05269\right)}^{2}+{\left(z-5.5\right)}^{2}\leq 0.24$ . In addition to that, for the one-dimensional simulations, we considered a domain *Ω* = [0, 8] cm, where the following boundary conditions were considered: u=0 at *x* = 0, traction free at *x* = 8, and no-flux boundary conditions for *c*_*p*_, *c*_*l*_, and *p* at *x* = 8.

**TABLE 1:** Reference parameters values used for experiments in Section 3.

Parameter	Description	Value (Unit)
*E*	Young modulus	60 (*kg*/*cm s* ^2^)
*Ν*	Poisson coefficient	3.5 × 10^–1^ (−)
*ρ*_*f*_	Fluid phase density	1 × 10^–3^ (*kg*/*cm*3)
*ρ*_*s*_	Solid phase density	2 × 10^–3^ (*kg*/*cm*3)
*Α*	Biot modulus	2.5 × 10^–1^
*d*_*p*_	Pathogen diffusion coefficient	See Table 2
*d*_*l*_	Leukocyte diffusion coefficient	See Table 2
*Χ*	Chemotaxis	1 × 10^–2^ (*cm* ^*2*^/*d* (*cell*/*cm* ^*3*^))
*γ*_*p*_	Pathogen reproduction rate	See Table 2
*λ*_*lp*_	Phagocytosis rate	1.5 (1/*d* (*cell*/*cm* ^*3*^))
*λ*_*pl*_	Leukocytes migration rate	See Table 2
*π*_*i*_	Interstitial oncotic pressure	10 (*mmHg*)
*π*_*c*_	Capillary oncotic pressure	20 (*mmHg*)
*σ*_0_	Osmotic reflection coefficient	9.1 × 10^−1^
*L*_*bp*_	Pathogen influence on permeability	1 × 10^4^ (1/(*cell*/*cm* ^3^))
*P*_*c*_	Capillary pressure	20 (*mmHg*)
*L*_*p*0_	Hydraulic permeability	3.6 × 10^–8^ (*cm*/*s mmHg*)
*q*_0_	Normal lymphatic flow	6.82 × 10^–5^ (1/*s*)
*k*_*m*_	Half life of lymphatic flow	6.5 (*mmHg*)
*N*	Hill coefficient	1
*V*_max_	Max lymphatic flow	200
*κ*_0_	Permeability	2.5 × 10^**–7**^ (*cm* ^2^/*s mmHg*)
*μ*_*f*_	Dynamic viscosity	1 (*cm* ^2^/*s*)
(*S*/*V*)	Vessel area per volume unit	174 (1/*cm*)
*ϕ*_0_	Initial fluid phase	2 × 10^–1^

**TABLE 2:** Parameter values used to represent local and diffuse myocarditis for the 1D and 3D simulations.

Parameter	Unit	Value	Diffuse 1D	Local 3D	Diffuse 3D
		Local 1D			
*d*_*p*_	*cm* ^2^/*d*	1 × 10^–3^	5 × 10^–3^	5 × 10^–4^	1 × 10^–3^
*d*_*l*_	*cm* ^2^/*d*	5 × 10^–2^	5 × 10^–2^	5 × 10^–1^	3 × 10^–2^
*λ*_*pl*_	1/*d* (*cell*/*cm* ^3^)	2.1 × 10°	1 × 10^–1^	1.2 × 10^–1^	5 × 10^–2^
*γ*_*p*_	1/*d*	9.0 × 10^–2^	3 × 10^–2^	9.0 × 10°	6 × 10^–2^

### 3.1 Immune System and Poroelastic Dynamics Over Time

**FIGURE 2:**
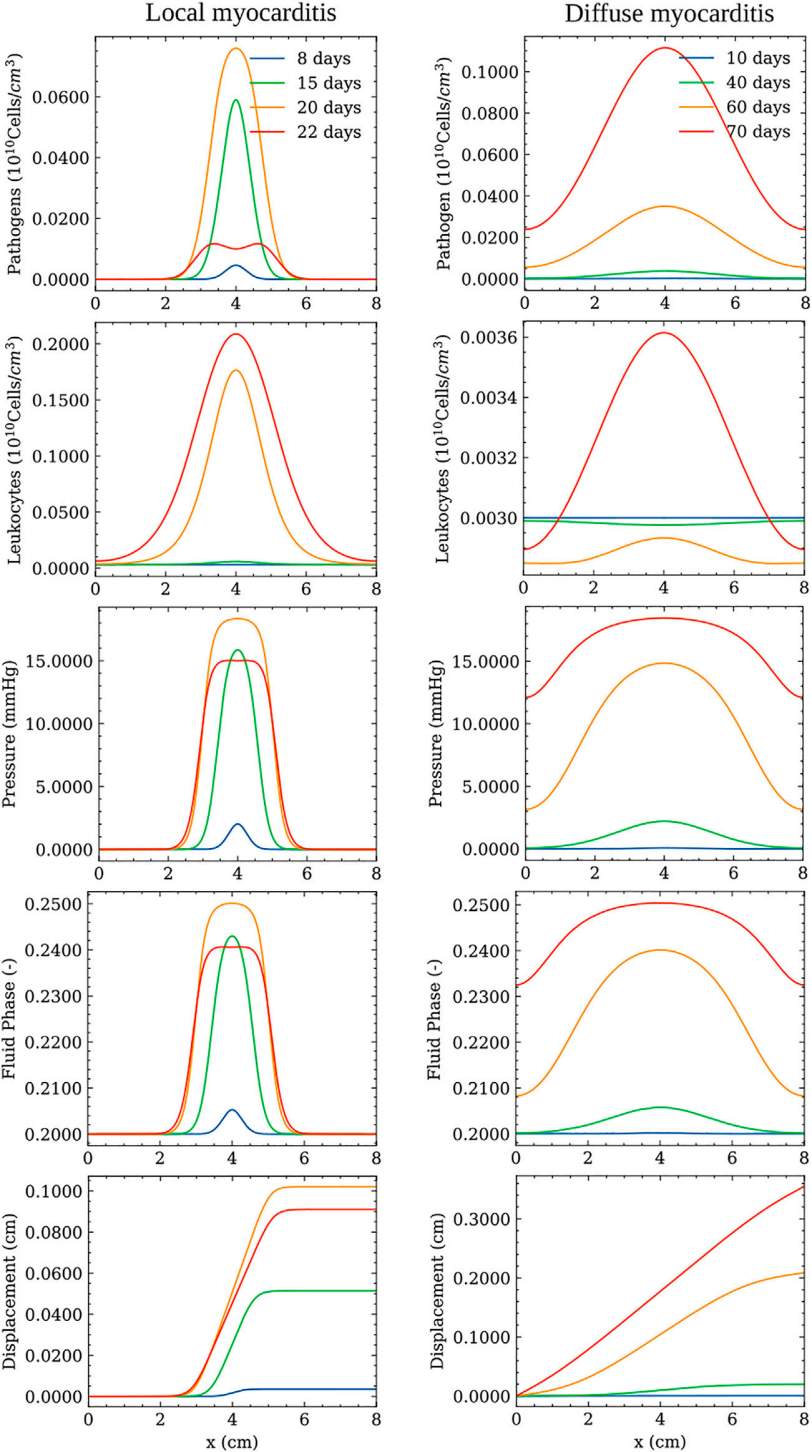
Simulation of local (left) and diffuse (right) myocarditis dynamics in a one-dimensional domain using the reference parameter values described in Table 1. Pathogen concentration, leukocytes concentration, pressure, displacement, and fluid phase fields are presented at different time instants after the onset of infection.

This section presents the results of the dynamics of pathogens, leukocytes, pressure, fluid phase, and displacement over the oedema formation. For the sake of simplicity, these results were obtained by simulating the model in a one-dimensional domain. Figure 2 presents the dynamics for local and diffuse myocarditis. Each figure presents the values of the five variables of the model over the spatial domain taken at four different time instants. These time instants are different because of the distinct nature of local and diffuse myocarditis. For the same reason, the values of some of the parameters are different. In particular, the pathogen diffusion rate (*d*_*p*_), leukocytes migration rate from the bloodstream (*λ*_*pl*_), and pathogen’s reproduction rate (*γ*_*p*_) had their values changed from the reference values (Table 1) to obtain the diffuse myocarditis pattern. The modified values used in the simulations are presented in Table 2.

### 3.2 Myocardial Oedema in the Human Left Ventricle

**FIGURE 3:**
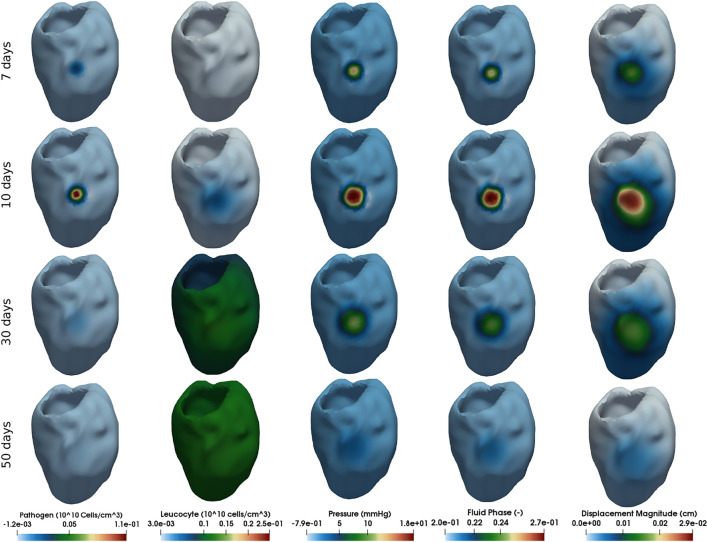
Simulation of local myocarditis in a 3D patient-specific heart geometry using the reference parameter values described in Tables 1, 2 (those from column Local Myocarditis). The rows present snapshots of the solution at different time instants (7, 10, 30, and 50 days) for each variable (columns).

**FIGURE 4:**
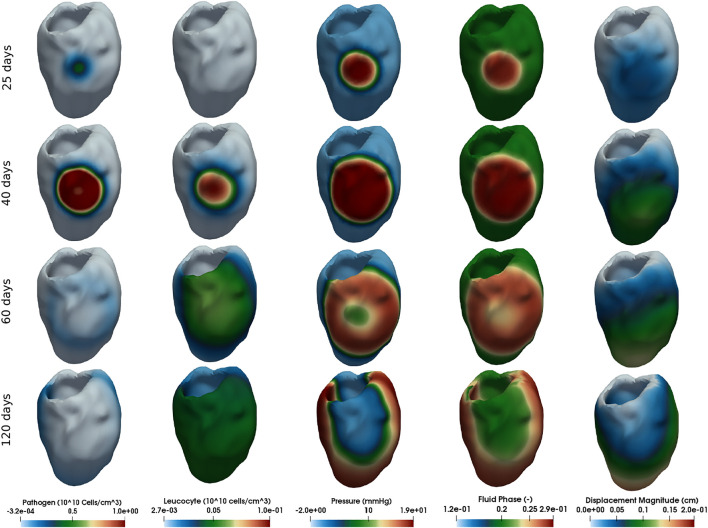
Simulation of diffuse myocarditis in a 3D patient-specific heart geometry using the reference parameter values described in Tables 1, 2 (those from column Diffuse Myocarditis 3D). The rows present snapshots of the solution at different time instants (25, 40, 60, and 120 days) for each variable (columns).

This section presents the myocardial oedema in a the human LV, considering two scenarios: local and diffuse myocarditis. Figures *dp*3, 4 present the solution of Eqs 2.3a, 2.3b, 2.3c, 2.6a, and 2.6b considering a ventricular geometry segmented from patient-specific images (Warriner et al., 2018). Figure 3 presents the results of a myocarditis simulation in which oedema stays localised only in part of the LV. Figure 4 reproduces a scenario of diffuse myocarditis, *i.e.*, oedema spreads all over the heart.

Each column of Figures 3, 4 represent one variable of interest: pathogen concentration, leukocyte concentration, displacement, pressure and phase. Each line present, in a distinct point in time, the values of these variables.

### 3.3 Sensitivity Analysis

**FIGURE 5:**
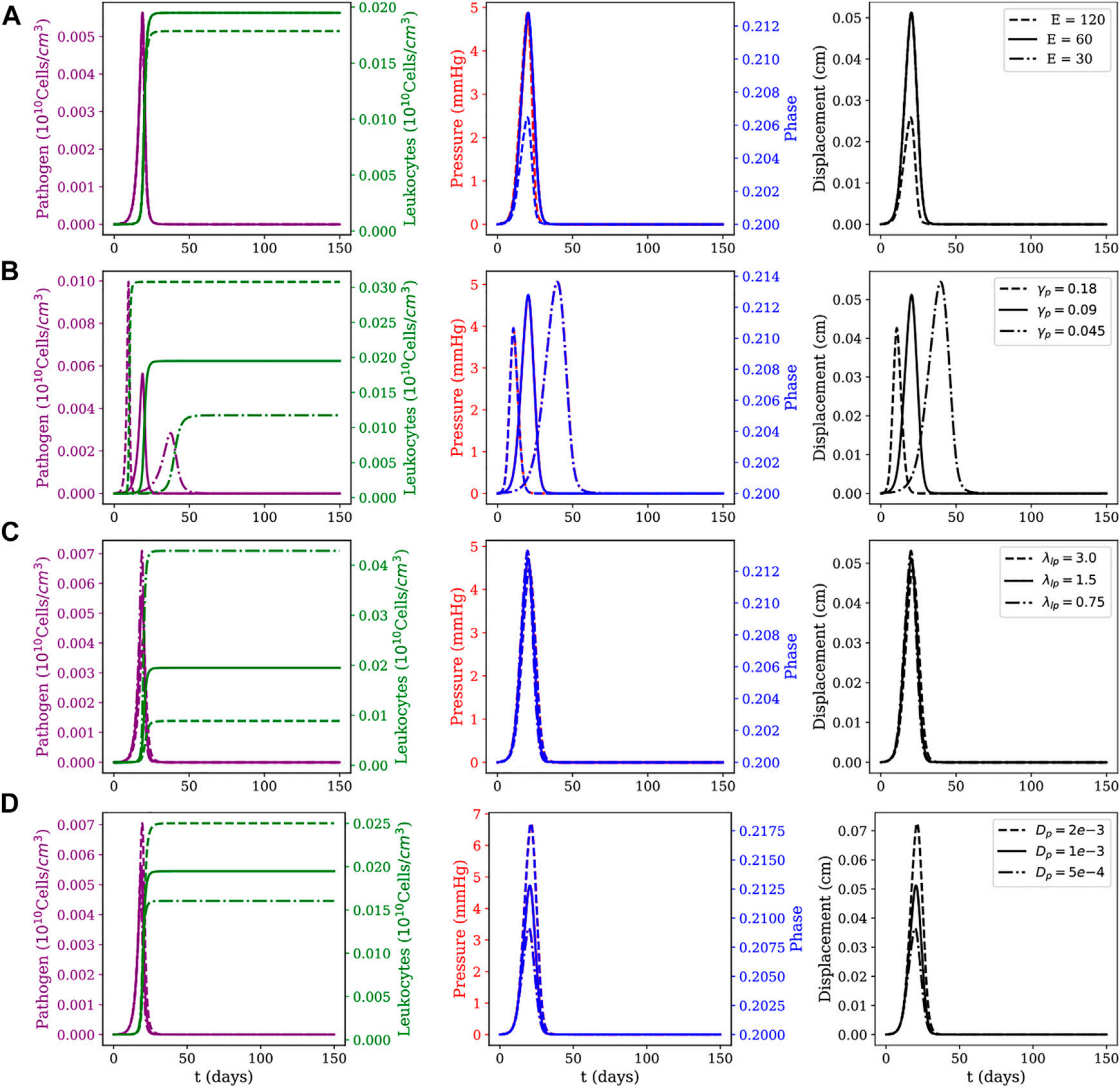
Local myocarditis: evolution of pathogens and leukocytes concentration, pressure, fluid phase, and displacement over 150 days of simulation for different parameter sets. Effects of variations on **(A)** Young modulus *E* (as computed from the Lamé parameters for the neo-Hookean model), **(B)** pathogen reproduction rate *γ*_*p*_, **(C)** phagocytosis rate *λ*_*lp*_; and **(D)** pathogens’ diffusion coefficient *d*_*p*_. For each row, three scenarios for the parameter under study are considered: one using the reference value described in Table 1, one using its value doubled and one using its value halved.

**FIGURE 6:**
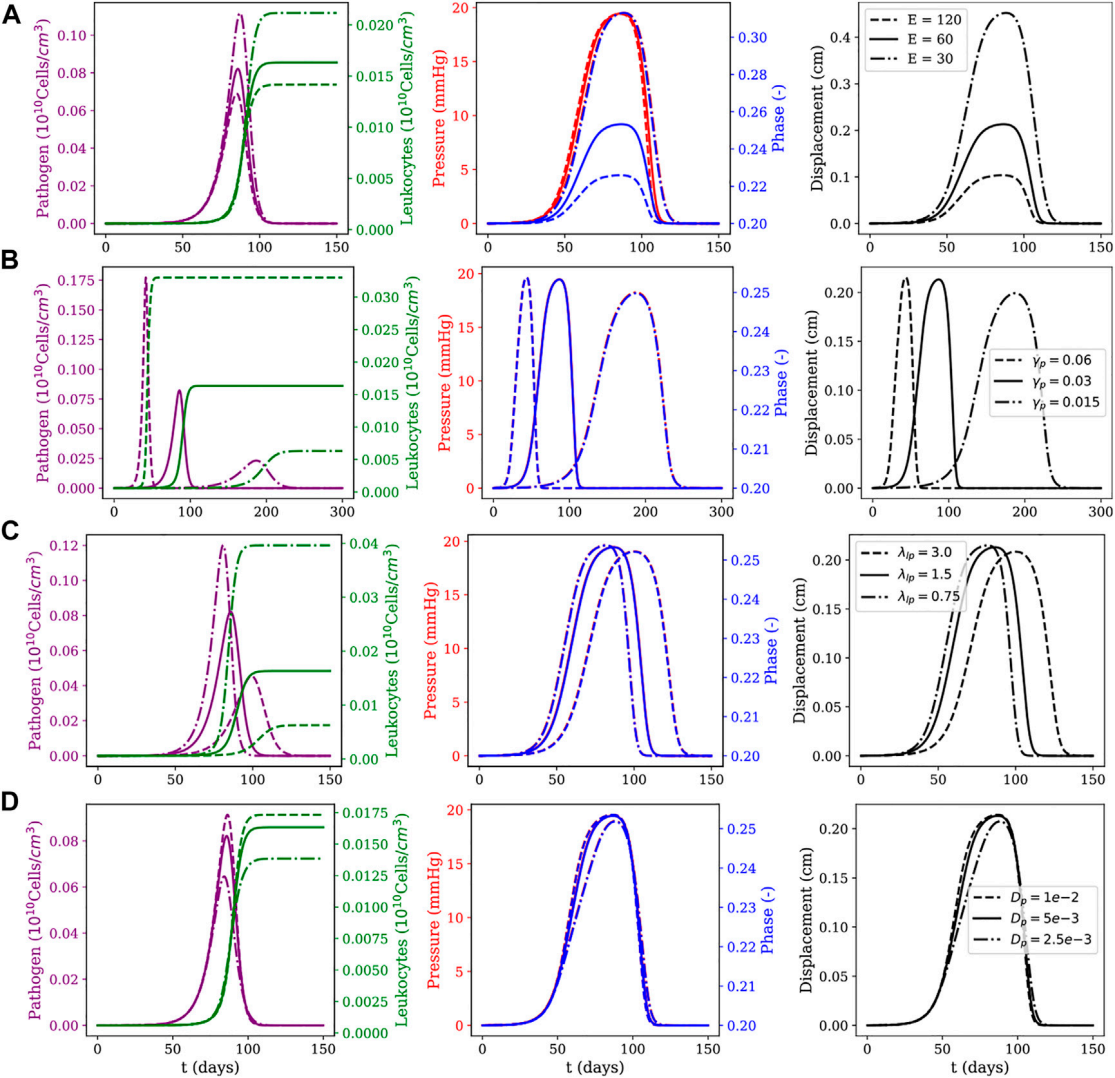
Diffuse myocarditis: evolution of pathogens and leukocytes concentration, pressure, fluid phase, and displacement over 150 days of simulation for different parameter sets. The first row **(A)** shows the effects of Young modulus *E* (as computed from the Lamé parameters for the neo-Hookean model); the second row **(B)** shows the effects of the pathogen reproduction rate *γ*_*p*_; the third row **(C)** shows the effects of the phagocytosis rate *λ*_*lp*_; and the last row **(D)** shows the effects of the pathogens’ diffusion coefficient *d*_*p*_. For each row, three scenarios for the parameter under study are considered: one using the reference value described in Table 1, one using its value doubled and one using its value halved.

An one-at-a-time sensitivity analysis was performed for all parameters of the model for the 1D case. Figures 5, 6 show, in each line, the influence of some of the most relevant model parameters on the dynamics of the local and diffuse myocarditis, respectively, identified on this analysis to better highlight the couplings of the model. In Figures 5, 6, we present the mean values of the model variables over the one-dimensional spatial domain at each time step.

The sensitivity analysis presented in Figures 5, 6 considered the following parameters: Young’s modulus (*E*), pathogen’s reproduction rate (*γ*_*p*_), phagocytosis rate (*λ*_*lp*_), and pathogen diffusion coefficient (*d*_*p*_). These parameters are also related to critical biological responses depending on their values. Changes in the value of *γ*_*p*_, for example, can be associated with pathogens with distinct proliferation abilities; changes in the value of *λ*_*lp*_ can describe the ability of leukocytes in dealing with the invading pathogen; whereas changes in *E* represent tissues with different stiffness. The sensitivity analysis was done as follows: all three parameters (*E*, *λ*_*lp*_, and *γ*_*p*_) had their values doubled and halved with respect to the reference value reported in Table 1, generating three scenarios: one using the reference value, one using its value doubled and one using its value halved. For each scenario, all other parameters were kept with the values described in Table 1.

To study these three scenarios, we assume that pathogens are responsible for triggering the inflammatory response. This choice is reasonable, since all the dynamics occur only due to the presence of pathogens in the system. For this reason, the instant in which pathogens’ concentration reaches its peak is used as a reference to collect data from five variables of interest: pathogen concentration, leukocytes concentration, pressure field, phase, and tissue displacement field. The columns have two *y*-axis, representing two distinct variables of interest. In the first column, the left *y*-axis represents the pathogen concentration and the right *y*-axis represents the leukocytes concentration. In the second column, the left *y*-axis represents the pressure in the tissue and the right *y*-axis represents the phase. The last column represents the displacement only, and in all figures, the *x*-axis represents time.

## 4 Discussion

Table 2 presents the set of parameters changed to obtain a local or a diffuse myocarditis during 1D and 3D simulations. We consider a local myocarditis when a small portion of the tissue presents an oedema, and a diffuse one if the oedema is not limited to a single portion of the tissue, spreading across all the domain. For the 1D case, the values of pathogen diffusion (*d*_*p*_), leukocytes migration rate (*λ*_*pl*_) and pathogen reproduction rate (*γ*_*p*_) were changed, in relation to the local oedema, to obtain a diffuse oedema. For the 3D case, the values of leukocyte diffusion (*d*_*l*_), *λ*_*pl*_ and *γ*_*p*_ were changed. Observe that the last two parameters were changed in both 1D and 3D cases. All parameters selected to be changed in 1D and 3D cases where chosen based on empirical observations obtained during the sensitivity analysis. An exhaustive search was not performed but could systematically find all combinations of parameters that could give rise to diffuse myocarditis. It is worth highlighting that in the 1D local myocarditis case, the leukocytes migration rate (*λ*_*pl*_) is higher than in the diffuse myocarditis case. The difference in these rates can be related to the endothelium, which works as a physical barrier between the bloodstream and the tissue. The migration of leukocytes from the bloodstream to the tissue is the first step to combat the presence of a pathogen. Failures in the migration mechanism can negatively impact the inflammatory response and for this reason, has already been recognised as a target for many diseases (Ley and Reutershan, 2006). Moreover, to reproduce the 1D local myocarditis it was also necessary to change the value of the pathogen reproduction rate (*γ*_*p*_), adjusting it to be faster than in the case of the diffuse myocarditis. This setting suggests a pathogen with a faster reproduction due to its biological characteristics or due to favourable conditions (facility to enter cells, abundance of nutrients, optimal temperature, for example). Similar behaviour was observed in the 3D case.

The left column of Figure 2 shows the local myocarditis dynamics, where one can observe its local behaviour through the evolution of pathogen concentration, pressure, and fluid-phase spatial distributions. After the pathogen enters the tissue and reproduces/replicates, leukocytes start to grow, causing an increase in the fluid phase and pressure (we set *p* = 0 to represent the normal pressure in the tissue), impacting the tissue displacement. This dynamic is kept, and, after 20 days, the pathogen concentration achieves its peak. The pathogens do not spread across all the domain due to their interaction with the growing concentration of leukocytes. Therefore, pathogens are restricted to a small region in the centre of the domain. It is also important to note that the peak of other populations may occur after 20 days since we focus on presenting a snapshot of the dynamics when the pathogen achieves its peak value. At time *t* = 22 days, one can note that the pathogen’s spread is controlled by the leukocytes, which start to act by reducing the concentration of the pathogen, and also reflects through the couplings of the model to the pressure and fluid phase fields. Differently from the local myocarditis, the right column of Figure 2 shows diffuse myocarditis formation. Again, the presence of a pathogen in the tissue starts the same cascade of events. However, due to differences in parameter values, it is possible to observe that this time the pathogen spreads across all the domain, achieving both borders. This process occurs slowly, taking more than 60 days to occur. After 70 days, the pathogen concentration achieves its peak. As a result of pathogens located in all points of the domain, the fluid phase and pressure increase more than their counterparts in the local myocarditis case.

The literature reports that viral myocarditis in murine is usually eliminated within 2 weeks following infection (Caforio et al., 2013). For susceptible murine strains, the inflammation can persist for several weeks (Caforio et al., 2013). Numerical results, from a qualitative perspective, were able to reproduce this behaviour, as Figures 3, 4 show. The first column of Figure 3, which represents the local myocarditis, shows that the pathogen was almost eliminated in less than 30 days, although its effect in the heart persists for more days. In the case of leukocyte concentration, the population remains constant because we did not included, for simplification purposes, a natural decay rate to *c*_*l*_ population. In the case of the diffuse myocarditis, its is possible to observe that a pathogen wave, after 120 days, is still crossing the LV, recruiting leukocytes to the tissue and impacting the fluid phase, pressure and displacement.

The first line of Figures 5, 6 present the numerical results for varying the Young’s modulus (*E*), *i.e.*, the tissue stiffness. A higher value indicates a stiffer tissue, *i.e.,* a tissue with a lower capacity to deform and accumulate fluid. As one can observe, a higher value for *E* results in small changes in phase and displacement. This phenomenon is observed in the results of both figures, although it is more evident in the diffuse myocarditis, where the results obtained with *E* = 30 and *E* = 60 lead to distinct phase and displacement behaviours. In the case of local myocarditis, the use of *E* = 30 or *E* = 60 produce similar results for phase and displacement. Pressure achieves about the same peak value for all values of *E* in both figures. But in the diffuse myocarditis (Figure 6) low values of *E* seems to impact phase and displacement in the tissue for more days than high values. Finally, in the local myocarditis, the pathogen’s peak value was not affected by changes in *E*, but leukocyte’s peak concentration was affected. This occurs because an increase in pressure impairs leukocytes to enter the tissue. In the case of diffuse myocarditis, both pathogen’s peak value and leukocyte’s peak concentration were affected.

The second line of Figures 5, 6 present the impacts of changing the pathogen reproduction rate (*γ*_*p*_) in the dynamics of simulation. An increase in *γ*_*p*_ can be interpreted as a pathogen that can reproduce faster. In fact, as the first column of both figures show, the peak of infection was higher and faster for larger *γ*_*p*_ values. Again, a higher pathogen concentration translates into higher leukocyte concentration since more pathogens in tissue lead to more leukocytes migrating to it. This faster increase in the concentration of leukocytes contains the infection quickly, preventing pathogens from remaining in the tissue long enough to spread. The pathogen concentration also directly impacts pressure, phase, and displacement, especially in the local myocarditis scenario: the higher its concentration, the higher the impact on these three components. In the case of diffuse myocarditis, *γ*_*p*_ significantly affects the duration of the effects on pressure, phase, and displacement.

In the third line of Figures 5, 6 one can observe how the simulations are affected by changes in the phagocytosis rate (*λ*_*lp*_). A low *λ*_*lp*_ value can be related to an ineffective immune system, *e.g.*, due to immunodeficiency. The diffuse myocarditis scenario is the only affected: clearly, the concentration of pathogens reaches a higher peak of infection for smaller values of *λ*_*lp*_. The lower capacity of phagocytosis by leukocytes leads to an increase in the concentration of pathogens, which in turn induces an increase in the capillary permeability and allows more fluid to enter the tissue. This increase in phase is responsible for the slight increase in pressure. Both events are responsible for the deformation suffered by the tissue, which is slightly greater for higher values of *λ*_*lp*_. In the case of the local myocarditis, the only population affected is the leukocytes themselves.

Finally, the last line of Figures 5, 6 show the impact of the pathogen diffusion rate on the numerical results. The big picture in both figures are almost the same: if the pathogen can diffuse faster, it achieves higher concentration than if diffuses slowly. As a consequence, the leukocyte concentration is higher when the pathogen concentration is higher. Phase, pressure, and displacement in tissue are affected as usual.

In summary, our simulations highlight that both mechanical properties of the tissue, effectiveness of the immune response, and pathogen features dictate the dynamics of myocarditis and oedema formation. In particular, we have observed that small values of tissue stiffness, *E*, and the ratios *λ*_*lp*_/*γ*_*p*_ (phagocytosis/pathogen reproduction) and *d*_*l*_/*d*_*p*_ (leukocyte/pathogen mobility) facilitate the spread of both pathogen and oedema in the heart.

Although the mechanisms behind the formation of the global oedema were the same in both 1D and patient-specific geometries, the pattern formation was very distinct. In 1D, the pathogen could monotonically increase with time all over the domain. In contrast, the formation of global oedema in the patient-specific model followed the propagation of a 3D wave of pathogens that initiates from a small infected region (the initial condition) and travels throughout the cardiac tissue until it collides with itself and vanishes. The presence of pathogens induces the local entry of leukocytes and fluid into the interstitial space of the tissue. As the wave of pathogens sweeps the entire heart, we see the formation of global oedema. This pattern suggests the existence of a non-linear wave of pathogens as typically observed in complex reaction-diffusion mechanisms. The propagation of the front of pathogens likely depends on the diffusion and replication of pathogens. On the other side, the wave tail likely depends on the diffusion and efficiency of the leukocytes. However, the sensitivity analysis revealed that other tissue features could also impact the global oedema formation, such as tissue stiffness. Although we were able to identify the existence of this non-linear wave in the formation of global oedema using the new proposed model, its proper characterisation deserves further analysis in future studies.

We also plan to investigate, in the near future, how the myocardial fibre-sheet architecture affects oedema formation. In this case, replacing the neo-Hookean constitutive model used here with a more realistic one (Holzapfel and Ogden, 2009). This study has also neglected the effects of cardiac contraction. The relevance of the contraction of cardiac myocytes in oedema formation also deserves further attention in future studies, as well as: the investigation of other constitutive relations for the hydraulic conductivity; the inclusion of lymphatic sinks for *c*_*p*_ and *c*_*l*_; modification of the model to include leukocyte’s decay as well as other cells of the immune system, in particular, those related to the adaptive immune system (T and B cells, antibodies, etc.) as proposed in Reis et al. (2021); a more detailed description of the complex porous media (Alves et al., 2019), since here we focused only on the interstitial space between cells; and a more quantitative validation of the model against patient-specific data.

## 5 Conclusion

This work has proposed a poroelastic approach for modelling myocardial oedema in acute myocarditis. The model captured the phenomenological features that occur during the interaction between pathogens and the immune system, considering a saturated poroelastic medium that admits large deformations. The model consists of a set of coupled equations described in terms of displacement, fluid pressure, fluid phase, and concentrations for leukocytes and pathogens. A finite element method was used for its numerical solution. The model was successfully used to study the immune system and poroelastic dynamics over time and the formation of myocardial oedema in a geometry that represents the human LV obtained from patient-specific images. Local and diffuse myocarditis were generated in simplified and complex geometrical domains, such as a patient-specific model. Sensitivity analysis suggests that both mechanical properties of the tissue, the effectiveness of the immune response, and pathogen features dictate the dynamics of myocarditis and oedema formation. In particular, we have observed that small values of tissue stiffness and the ratios (phagocytosis/pathogen reproduction) and (leukocyte/pathogen mobility) facilitate the spread of pathogen and oedema in the heart. In the patient-specific model, we identified that the propagation of a non-linear wave of pathogens was behind the global oedema formation. Altogether, the results suggest that the new proposed model that couples tissue poroelasticity with the immune response is a powerful tool for studying myocarditis and oedema formation. Of particular interest is the support of mechanistic investigations of the different dynamics found in local and diffuse myocarditis.

## Data Availability

The datasets presented in this study can be found in online repositories. The names of the repository/repositories and accession number(s) can be found below: https://github.com/ruizbaier/PoroelasticModelForAcuteMyocarditis.
